# Benzimidazoisoquinolines: A New Class of Rapidly Metabolized Aryl Hydrocarbon Receptor (AhR) Ligands that Induce AhR-Dependent Tregs and Prevent Murine Graft-Versus-Host Disease

**DOI:** 10.1371/journal.pone.0088726

**Published:** 2014-02-19

**Authors:** Sumit Punj, Prasad Kopparapu, Hyo Sang Jang, Jessica L. Phillips, Jamie Pennington, Diana Rohlman, Edmond O’Donnell, Patrick L. Iversen, Siva Kumar Kolluri, Nancy I. Kerkvliet

**Affiliations:** 1 Department of Environmental & Molecular Toxicology, Oregon State University, Corvallis, Oregon, United States of America; 2 Environmental Health Sciences Center, Oregon State University, Corvallis, Oregon, United States of America; University of Texas at San Antonio, United States of America

## Abstract

The aryl hydrocarbon receptor (AhR) is a ligand-activated transcription factor that plays multiple roles in regulation of immune and inflammatory responses. The ability of certain AhR ligands to induce regulatory T cells (Tregs) has generated interest in developing AhR ligands for therapeutic treatment of immune-mediated diseases. To this end, we designed a screen for novel Treg-inducing compounds based on our understanding of the mechanisms of Treg induction by the well-characterized immunosuppressive AhR ligand, 2,3,7,8-tetrachlorodibenzo-*p*-dioxin (TCDD). We screened a ChemBridge small molecule library and identified 10-chloro-7H-benzimidazo[2,1-a]benzo[de]Iso-quinolin-7-one (10-Cl-BBQ) as a potent AhR ligand that was rapidly metabolized and not cytotoxic to proliferating T cells. Like TCDD,10-Cl-BBQ altered donor CD4^+^ T cell differentiation during the early stages of a graft versus host (GVH) response resulting in expression of high levels of CD25, CTLA-4 and ICOS, as well as several genes associated with Treg function. The Treg phenotype required AhR expression in the donor CD4^+^ T cells. Foxp3 was not expressed in the AhR-induced Tregs implicating AhR as an independent transcription factor for Treg induction. Structure-activity studies showed that unsubstituted BBQ as well as 4, 11-dichloro-BBQ were capable of inducing AhR-Tregs. Other substitutions reduced activation of AhR. Daily treatment with 10-Cl-BBQ during the GVH response prevented development of GVH disease in an AhR-dependent manner with no overt toxicity. Together, our data provide strong support for development of select BBQs that activate the AhR to induce Tregs for treatment of immune-mediated diseases.

## Introduction

The aryl hydrocarbon receptor (AhR) is a ligand-activated transcription factor that is gaining increasing attention as an important regulator of many aspects of immunological function. Activation of AhR has been implicated in the development of innate lymphoid cells in the gut [1], [2], induction of tolerogenic dendritic cells [3], and modulation of several aspects of T cell differentiation, including the induction of regulatory T cells (Tregs) [4], [5]. AhR-deficient mice are hyper-responsive to inflammatory stimuli, showing increased production of inflammatory cytokines and increased immunopathology [6], [7], supporting an immunoregulatory role for the AhR. Current evidence suggests that kynurenine, a product of tryptophan metabolism, is an endogenous AhR ligand that plays an integral role in the induction of peripheral tolerance [8], [9], while other exogenous AhR ligands such as 2,3,7,8-tetrachlorodibenzo-p-dioxin (TCDD) are among the most potent immunosuppressive chemicals known [10].

Our laboratory originally identified the AhR in CD4^+^ T cells as the direct, primary target for TCDD’s potent suppression of a murine acute graft versus host (GVH) response [11], [12]. An AhR-dependent Treg phenotype emerged during the initial activation and expansion of the naïve allospecific CD4^+^ donor T cells and was characterized by high expression of CD25 and CTLA-4 but no Foxp3, implicating AhR as a unique transcription factor driving Treg development [4]. These AhR-induced Tregs (AhR-Tregs) showed potent suppression of naïve and allogeneic T cell proliferation *in vitro* and increased expression of several genes that have been associated with other types of Tregs [4], [13]. The induction of AhR-Tregs that suppress the development of effector cytotoxic T lymphocytes (CTL) is consistent with the early window of susceptibility to immune suppression induced by TCDD. For example, if treatment with TCDD is delayed beyond day 3 of an allograft response, the alloCTL response is not suppressed [14]. In other disease models, activation of AhR has been associated with increased frequency of Foxp3^+^ Tregs [5], [15], [16]. These Foxp3^+^ Tregs emerge over several days or weeks in different autoimmune disease models and may develop indirectly via AhR-dependent induction of tolerogenic dendritic cells [3], [9]. Both types of Tregs likely contribute to control of autoimmune diseases following treatment with AhR ligands [4], [5], [15], [16].

Induction of immune tolerance by Tregs has generated considerable interest as an alternative therapy to conventional immunosuppressive treatment of autoimmune diseases that fail to provide long-term remission without severe side effects [17]. However, current clinical trial protocols that rely on the infusion of *in vitro*-derived polyclonal Tregs or *ex vivo*-expanded autologous Tregs are constrained by technical challenges and high cost [18]. In contrast, direct, pharmacological induction of Tregs in patients using nontoxic AhR ligands would provide the convenience of a drug to maximize treatment efficacy and minimize side effects.

In the current studies, we used our knowledge of TCDD-induced AhR-Tregs to screen a small molecule library for compounds that could activate AhR at nanomolar (nM) concentrations, were not cytotoxic to proliferating T cells *in vitro*, and induced the AhR-Treg phenotype *in vivo* [19]. Here we describe the characterization of a high affinity AhR ligand, 10-chloro-7H-benzimidazo[2,1-a]benzo[de]isoquinolin-7-one (10-Cl-BBQ), as a promising lead compound for clinical development. Unlike TCDD, 10-Cl-BBQ is rapidly metabolized yet is capable of inducing AhR-dependent Tregs and suppressing the murine graft versus host disease (GVHD) in an AhR-dependent manner. Structure-activity studies also identified two analogues of 10-Cl-BBQ that induce AhR-Tregs. Overall, our results support the pursuit of this new class of AhR ligands for Treg-based immunotherapy.

## Materials and Methods

### Mice

C57Bl/6 (H-2^b/b^), B6D2F1 (H-2^b/d^) and B6.129-AHR^tm1Bra^/J (AhR^−/−^) mice were obtained from The Jackson Laboratory. The AhR^−/−^ mice were bred and maintained in a specific pathogen-free vivarium at Oregon State University (Corvallis, OR). OSU is an AALAC-accredited institution. *Ethics Statement:* All experimental procedures and treatments using animals (mice) were approved by the Institutional Animal Care and Use Committee at Oregon State University. Euthanasia was by CO_2_ overdose followed by cervical dislocation.

### Identification and Characterization of AhR Ligands

**Figure 1:**
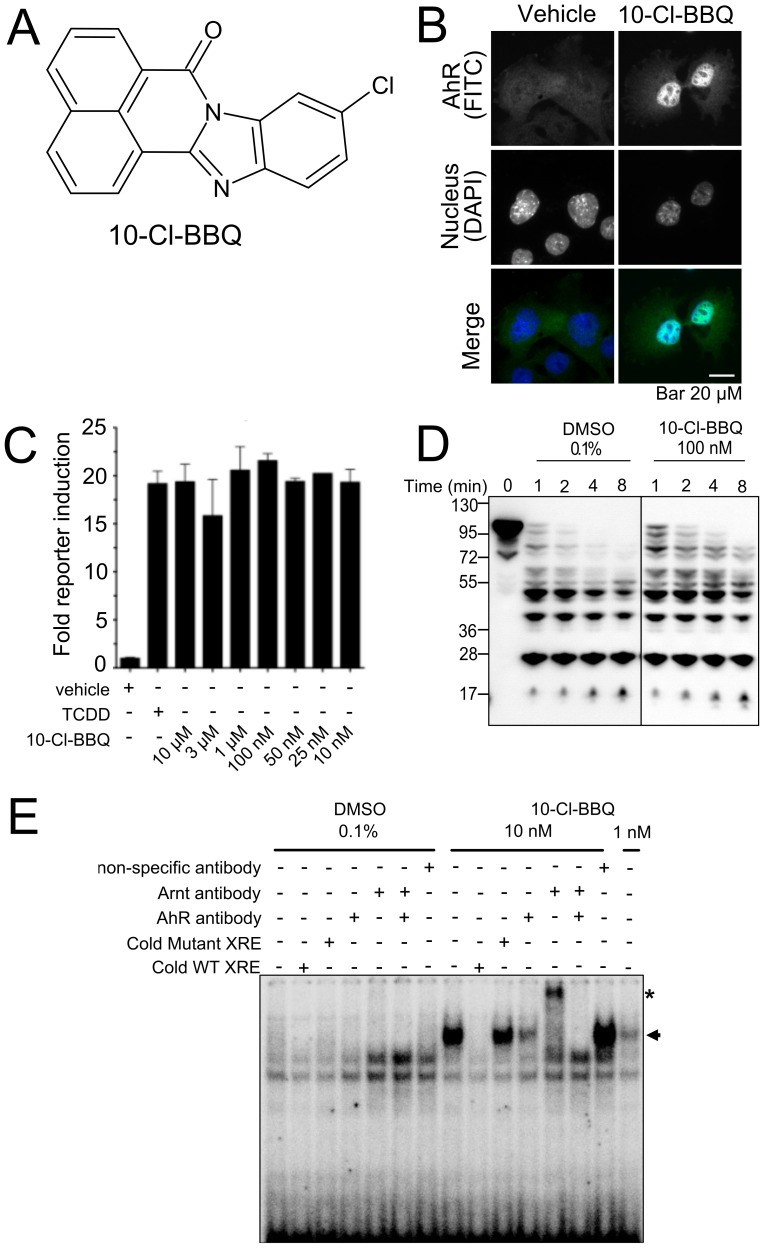
10-Cl-BBQ is a potent AhR ligand. **A)** Structure of 10-Cl-BBQ. **B)** 10-Cl-BBQ (10 nM) promoted cytosol to nuclear translocation of AhR after 1 h treatment of Hepa-1 cells. **C)** Hepa-1 cells expressing endogenous AhR were transfected with an AhR-regulated luciferase reporter gene (xenobiotic responsive element (XRE)-MMTVpromoter-Luc) and the reporter gene activity was measured after 12 h of treatment with 1 nM TCDD or the indicated concentrations of 10-Cl-BBQ. **D)** Alteration in proteolytic pattern of AhR by 10-Cl-BBQ. Whole cell lysate (45 µg of protein) from mouse hepatoma Hepa1c1c7 cells was incubated with 100 nM 10-Cl-BBQ or 0.1% DMSO for 1 h at RT and proteolyzed by 1 µg/ml subtilisin for 1, 2, 4, and 8 min at RT. Proteolytic cleavage products of AhR were analyzed by immunoblot. **E)** Electrophoretic mobility shift assay showing the ligand-stimulated binding of AhR to XRE. Whole cell lysate (18 µg of protein) from mouse hepatoma Hepa1c1c7 cells was incubated with 10 nM 10-Cl-BBQ or 0.1% DMSO for 2 h at room temperature and ^32^P-labeled oligonucleotide containing XRE for 20 min at room temperature. The samples were electrophoresed on a native polyacrylamide gel and the signal was visualized by Phosphor Imager. Unlabeled competitor XRE or antibody (Ab) against AhR or Arnt was co-incubated with ^32^P-labeled oligonucleotide. Arrow indicates specific signal and asterisk (*) shows the supershifted complex.

The DIVERSet library (ChemBridge) with 50,000+ compounds was used for primary screening. The AhR reporter gene assay was performed as previously described (20–22). DNA binding activity, nuclear localization, competitive binding and the electrophoretic mobility shift assays were conducted as previously described [20]–[23]. Experimental details are included in the legend of Fig. 1.

### Pharmacokinetic Studies

C57Bl/6 mice were injected i.p. with 10 mg/kg 10-Cl-BBQ. At defined time points, blood was collected by cardiac puncture. Serum was harvested and protein was precipitated by adding a 4-fold volume of ice-cold acetonitrile. The supernatant was collected and analyzed on a QTRAP 4000 LC/MS/MS instrument using an Agilent C8 UPLC column. The serum concentration of 10-Cl-BBQ was determined using a standard curve prepared using dilutions of 10,000; 5,000; 1,000; 500; 100; 50 and 10 pg/ml of 10-Cl-BBQ. The mean values (n = 2–3 mice per time point) are shown with SEM. Pharmacokinetic parameters were calculated using PKsolver software [24].

### Graft-versus-host Disease (GVHD) Model

Splenocytes and lymphocytes were harvested from C57Bl/6 mice. In some experiments, T cells were purified by negative selection using a Pan-T kit (Miltenyi Biotec) and stained with 2 µM carboxyfluorescein succinimidyl ester (CFSE) (Life Technologies). At least 2×10^7^ purified T cells or approximately 1×10^8^ pooled splenocytes and lymphocytes were injected i.v. into B6D2F1 host mice to initiate the GVH response. GVHD pathology was assessed on a scale of 0 to 3 (0 = normal, 1 = moderate morbidity, 2 = severe morbidity, 3 = death) according to the following five criteria: weight-loss, posture, activity, fur texture and death and reported as a Clinical GVHD Score.

### Treatment with TCDD or 10-Cl-BBQ

Host mice were treated daily with vehicle, 10-Cl-BBQ or BBQ analogues by i.p. injection. The vehicle consisted of a mixture of 6.7% anisole and peanut oil. Mice treated with a single i.p. dose of 15 µg TCDD/kg body weight in the same vehicle were used as positive controls.

### Flow Cytometry

Antibodies to CTLA-4, Foxp3, ICOS, CD62L, CD25, CD19, CD44) and a fixable viability dye were purchased from eBiosciences. Additional antibody reagents included CD4 and CD8 (Life Technologies); H-2D^d^ (Biolegend) and CD45RB (PharMingen). After surface staining, the cells were fixed and permeabilized with Cytofix/cytoperm (BD Biosciences) for intracellular CTLA-4 stain. The flow cytometry data were acquired on an FC500 instrument (Beckman Coulter) and the data were analyzed using WinList 7.0 (Verity Software House). Fluorescence minus one (FMO) controls were used for setting histogram gates during the analysis.

### RNA Isolation and Reverse Transcriptase Quantitative Polymerase Chain Reaction (RT-qPCR)

RNA was isolated from lymph nodes of B6D2F1 host mice with the RNeasy Mini Kit (Qiagen). cDNA was prepared from at least 1 µg RNA in a 2-step reaction using the ReactionReady First Strand cDNA synthesis kit (SA Biosciences) according to the manufacturer’s instructions and Superscript III reverse-transcriptase (Life Technologies). The qPCR reaction was carried out on an ABI PRISM 7500 Real-Time PCR system (Applied Biosystems). The reaction mixture consisted of SYBR Green/ROX qPCR Master Mix (SA Bioscience), 2–10 ng-RNA equivalent cDNA and 1 µl of the gene-specific commercial primer sets (SA Biosciences). The following conditions were used: 10 min at 95°C (1 cycle), 15 s at 95°C and 1 min at 60°C (40 cycles). The melt-curve was generated at 95°C for 15 s. The process was validated by utilizing a no-reverse-transcriptase control. The raw data was normalized to *Actb* and the fold-change was calculated by the ΔΔCt method using vehicle-treated sample values as the control.

### Statistics

All statistical analyses were performed using Prism (GraphPad Software). Treatment means were compared by one-way ANOVA followed by Dunnett’s post-test for multiple comparisons. A statistical significance of p<0.05 was used. The graphs are shown as mean values with standard error of mean (SEM) or Standard deviation (SD) as indicated.

## Results

### Identification and Characterization of 10-Cl-BBQ as an AhR Ligand

To identify novel AhR ligands, we screened the ChemBridge DIVERSet™ small-molecule chemical library. The initial screening system that we established was based on a double hybrid technique that was designed to find and/or characterize protein-protein interactions. We engineered AhR such that it activated a heterologous response element-driven luciferase reporter gene upon binding to a ligand. Our screening system enabled us to identify compounds that bind and induce a transcriptionally-active conformation of AhR. We identified 10-Cl-BBQ as a high affinity AhR ligand (Fig. 1A) and selected it for further characterization. 10-Cl-BBQ promoted cytosol to nuclear translocation of AhR (Fig. 1B) and activated the AhR-regulated reporter gene (Fig. 1C) at nanomolar concentrations. The compound specifically induced DNA binding of AhR and delayed its proteolysis (Fig. 1D and 1E). In hepatocytes, 10-Cl-BBQ induced a battery of genes that are known to be associated with AhR activation by TCDD (Fig. S1) [24]. 10-Cl-BBQ did not inhibit proliferation of activated T cells *in vitro* or *in vivo* (Fig. S2), nor did it induce acute hepatic toxicity in mice (Table S1). Overall, these results indicate that 10-Cl-BBQ is a high-affinity ligand and potent activator of the AhR.

### Pharmacokinetics of 10-Cl-BBQ

**Figure 2:**
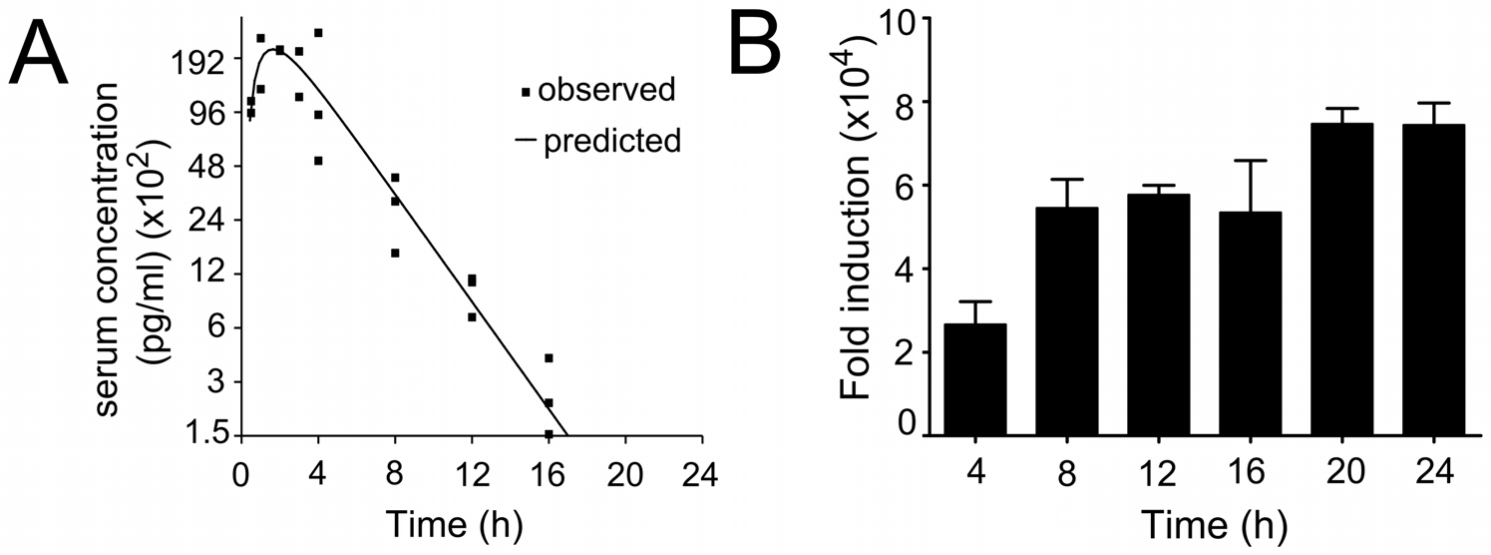
Pharmacokinetics of 10-Cl-BBQ. C57BL/6 mice were given 10 mg/kg 10-Cl-BBQ by i.p. injection. **A)** Blood was collected at the indicated times by cardiac puncture. The serum was processed to remove protein and the samples were analyzed by QTRAP 4000 LC/MS/MS. The serum concentration of 10-Cl-BBQ was determined from a standard curve based on known concentrations of 10-Cl-BBQ. **B)** Liver tissue was harvested and processed for mRNA analysis. CYP1A1 gene expression was determined relative to the β-actin gene, and the fold-induction was determined by the ΔΔCt method using the vehicle-treated samples as a control. The mean values are shown with SEM for 2–3 mice per time point.

TCDD is an extremely potent and long-acting immunosuppressive chemical. The extended action of TCDD results from its resistance to metabolic breakdown especially by cytochrome P450 monoxygenase enzymes (e.g., CYP1A1, CYP1B1) that are induced by AhR activation [23]. The long-half life of TCDD results in prolonged activation of the AhR, which has been postulated to play a role in the toxicity of TCDD. Thus, AhR ligands that are developed for therapeutic use must have more favorable pharmacokinetic properties than TCDD. To determine the half-life of 10-Cl-BBQ, we treated mice with a single intraperitoneal injection of the chemical, and measured serum concentrations over time by mass spectrometry. As shown in Fig. 2A, the data fit a one compartment model with first-order kinetics. Based on this model, the serum half-life of 10-Cl-BBQ is approximately 2 h. Additional kinetic parameters for absorption rate constant (*k_a_*); the maximum concentration reached in serum (C_max_) and the time required to reach C_max_ (T_max_), were 1.2 h^−1^, 21.51 µg/L and 1.64 h respectively. The area under the concentration-time curve (AUC_0-∞_) was 102.5 µg/L*h. The volume of distribution/bioavailability (V/F) and total clearance/bioavailability (CL/F) constants were 5.66 L and 32.5 mL/min respectively.

To assess the duration of activation of AhR in response to a single 10-Cl-BBQ treatment, the temporal induction of CYP1A1 mRNA was determined in hepatic tissue. As shown in Fig. 2B, *Cyp1a1* was induced 2-fold at 4 hours and up to 7-fold by 20 hr. Induction was no longer apparent at 48 h (data not shown). These data indicate that although the serum half-life of 10-Cl-BBQ is short, the extended induction profile of CYP1A1 mRNA suggests that there is tissue retention of the compound and that a once-a-day dosage regimen is sufficient to sustain AhR activation.

### Induction of AhR-Tregs by 10-Cl-BBQ

**Figure 3:**
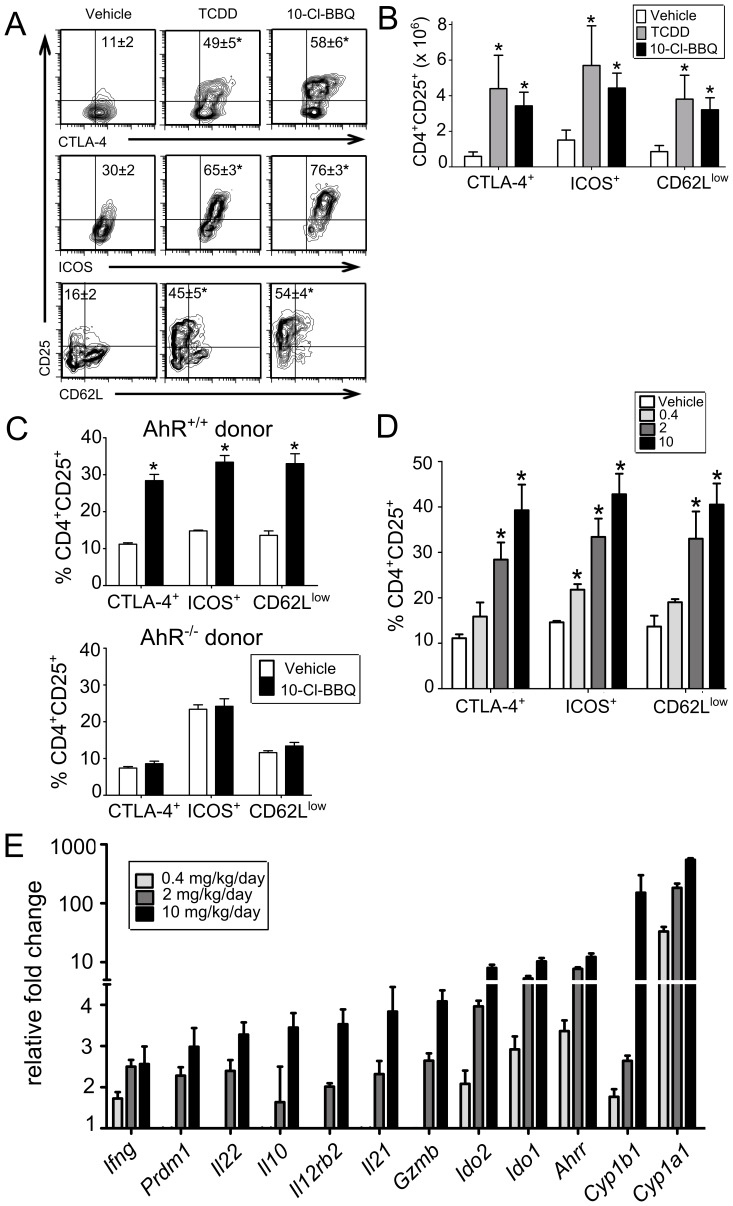
10-Cl-BBQ induces an AhR-dependent Treg phenotype 48 h after initiating the GVH response. On day 0, B6D2F1 host mice in groups of 5 were injected with CFSE-labeled C57Bl/6 T cells and given 15 mg/kg 10-Cl-BBQ, 15 µg/kg TCDD or vehicle by i.p. injection. At 24 h, an additional dose of 7 mg/kg 10-Cl-BBQ or vehicle was given. **A)** Histograms reveal the Treg phenotype based on coexpression of CD25 with CTLA-4, ICOS and down-regulated CD62L (CD62L^lo^) on alloactivated CD4^+^ donor T cells from each treatment group. Treatment mean ± SEM is shown in the quadrant of interest. **B)** The total number of alloactivated CD4^+^CD25^+^ donor T cells co-expressing CTLA-4^+^, ICOS^+^ and CD62L^low^ was also increased by treatment with 10-Cl-BBQ. **C)** AhR-dependent induction of the donor Treg phenotype by 10-Cl-BBQ. On day 0, B6D2F1 host mice in groups of 5 were injected with donor T cells obtained from AhR^+/+^ or AhR^−/−^ C57Bl/6 mice. Host mice were treated with 2 mg/kg10-Cl-BBQ or vehicle immediately following donor cell transfer and again at 24 hr. Treg phenotype analyzed on day 2 was significantly increased by 10-Cl-BBQ treatment when AhR^+/+^ donor T cells were injected but not when AhR^−/−^ donor T cells used. **D)** Dose-dependent induction of Treg phenotype by 10-Cl-BBQ. On day 0, B6D2F1 host mice in groups of 3–5 were injected with C57Bl/6 T cells. Mice were immediately treated with 0, 0.4, 2 or 10 mg/kg 10-Cl-BBQ and again on day 1. On day 2, expression of markers associated with the AhR-Treg phenotype increased as the dose of 10-Cl-BBQ increased. **E)** In the same mice as in D, lymph nodes were harvested and analyzed for expression of genes that were previously shown to increase under the same GVH conditions with TCDD treatment (13). Results show a similar profile of gene expression that is induced by treatment with 10-Cl-BBQ. Changes in gene expression were calculated relative to the β-actin gene, and the fold-induction was calculated by the ΔΔCt method using the vehicle-treated samples as a control. * = p<0.05 relative to vehicle treatment.

Treatment of mice with TCDD during the initiation of the murine parent-to-F1 GVH response induces donor-derived Tregs within 48 h by a mechanism that is mediated by the AhR in the donor CD4^+^ T cells [4], [12]. The AhR-induced Tregs were identified as the CD4^+^CD25^+^CTLA-4^+^CD62L^low^ population of T cells [4]. To determine if 10-Cl-BBQ induced similar phenotypic changes in donor CD4^+^ T cells, B6D2F1 (H-2^b/d^) host mice were injected with purified, CFSE-labeled donor C57Bl/6 (H-2^b/b^) T cells and treated with 15 mg/kg 10-Cl-BBQ by intraperitoneal injection. A second dose of 7 mg/kg was given one day later. Mice that were treated with TCDD at the time of donor cell transfer were used as positive controls. Splenocytes were isolated on day 2 for phenotypic analysis of the alloresponsive (CFSE-diluted) donor CD4^+^ T cells (Fig. S3). Compared with vehicle treatment, 10-Cl-BBQ significantly increased the percentage of donor CD4^+^ T cells that co-expressed CD25 and CTLA-4, along with low expression of CD62L (Fig. 3A). Expression of inducible T-cell co-stimulator (ICOS/CD278), another marker associated with Tregs [25], was also significantly increased by treatment with 10-Cl-BBQ as well as TCDD (Fig. 3A). Daily treatment with 10-Cl-BBQ induced the Treg phenotype as effectively as the known immunosuppressive dose of TCDD (15 µg/kg) that has been used in many prior studies [10]. The total number of CD4^+^ CD25^+^ T cells expressing CTLA-4, ICOS and low levels of CD62L was increased by both AhR ligands (Fig. 3B). Importantly, 10-Cl-BBQ failed to induce the Treg phenotype when donor T cells were obtained from AhR-deficient mice (Fig. 3C), demonstrating that the induction of the Treg phenotype by 10-Cl-BBQ was mediated via the AhR in the donor T cells. Prior studies have shown that the AhR in the CD4^+^ T cell is the primary target driving the AhR-Treg phenotype [12].

To determine a minimum effective dose of 10-Cl-BBQ to induce Tregs, we treated mice with 0, 0.4, 2 or 10 mg/kg/day for two days after initiation of the GVH response. Splenocytes were harvested at 48 h for analysis of the Treg phenotype and lymph nodes were processed for analysis of gene expression. Induction of the Treg phenotype increased with increasing dose of 10-Cl-BBQ (Fig. 3D) and generally correlated with degree of activation of AhR as reflected by *Cyp1a1, Cyp1b1* and *Ahrr* expression in lymph nodes from the same mice (Fig. 3E). Expression of several other genes previously shown to be up-regulated in lymph node cells (host- and donor-derived) of TCDD-treated mice [13], were also induced by 10-Cl-BBQ, including *Il10*, *Il21*, *Il22*, *Il12rb2*, *Gzmb, Ido1* and *Ido2*. The dose of 2.0 mg/kg/day was the minimum effective dose as 0.4 mg/kg/day failed to induce significant changes in most of the parameters associated with AhR-Tregs (Fig. 3D and 3E). Notably, like TCDD, 10-Cl-BBQ did not induce expression of *Foxp3* at any dose, and thus, in contrast to other reports [5], *Foxp3* does not appear to be a direct, AhR-inducible target gene in activated T cells *in vivo*.

### Suppression of Murine GVHD by 10-Cl-BBQ

**Figure 4:**
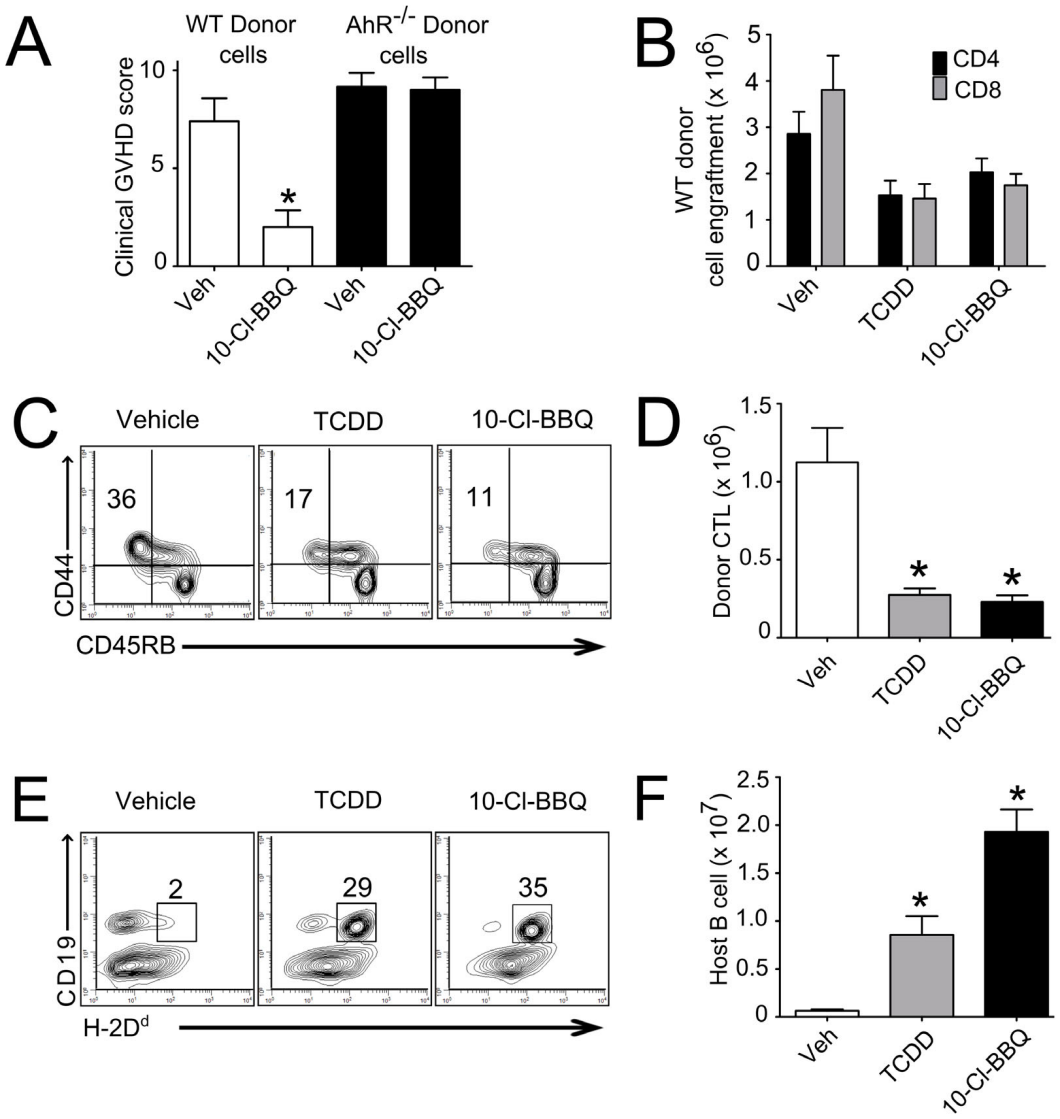
10-Cl-BBQ suppresses GVHD in an AhR-dependent manner. **A)** B6D2F1 host mice were injected with wild-type (WT) or AhR-deficient (AhR^−/−^) splenocytes to initiate the GVH response; mice were given 10-Cl-BBQ or vehicle treatments daily for 15 days. A single loading dose of 10 mg/kg 10-Cl-BBQ was given at the time of donor cell injection and a maintenance dose of 2 mg/kg was given every day thereafter until the end of the experiment. The progression of GVHD was monitored and a clinical pathology score as defined in Methods was calculated. GVHD was suppressed by 10-Cl-BBQ only if the donor cells expressed AhR. In a separate study, B6D2F1 host mice in groups of 6 were injected with splenocytes from C57Bl/6 mice to initiate the GVH response, and treated with 10-Cl-BBQ or vehicle daily for 15 days as in (A). TCDD (15 µg/kg) was given to an additional group of 6 mice on day 0 as a positive control. GVHD was significantly suppressed by 10-Cl-BBQ and TCDD as evidenced by **B)** reduced total number of donor CD4^+^ and CD8^+^ T cells engrafted in the host spleen at day 15, **C)** reduced percentage and (**D**) number of activated donor CD8^+^ T cells expressing a CTL phenotype (CD8^+^CD44^hi^CD45RB^low^), as well as (**E**) increased percentage and (**F**) number of host B cells (CD19^+^ cells) in the spleen at day 15. The donor cells were identified in the host spleen as the H-2D^d^ negative population.* = p<0.05 vs vehicle control.

Since 10-Cl-BBQ was capable of inducing AhR-Tregs, it was of interest to determine if it was also capable of suppressing GVHD, and if this suppression was dependent on AhR expression in the donor cells as was seen with TCDD [11]. GVHD was established using donor cells obtained from wild-type or AhR-deficient mice. Due to a limited number of AhR-deficient mice available, whole splenocytes rather than purified T cells were used to initiate the GVH response and no TCDD-treated positive control group was included. Host mice were treated on day 0 and 1 with 10 mg/kg 10-Cl-BBQ followed by 2 mg/kg/day until termination of the experiment on day 15. At termination, each animal was evaluated and given a clinical pathology score based on changes in weight, posture, activity, fur texture, and mortality. As shown in Fig. 4A, GVHD pathology was significantly reduced by treatment with 10-Cl-BBQ in mice that received AhR-wild type donor cells whereas the pathology score was not affected by 10-Cl-BBQ in mice that received AhR-deficient donor cells. These results document the AhR-dependent suppression of GVHD by 10-Cl-BBQ. In a separate study, the effect of 10-Cl-BBQ on donor cell engraftment was examined and directly compared with the effect of TCDD. As shown in Fig. 4B, 10-Cl-BBQ significantly reduced the engraftment of donor cells as well as the percentage (Fig. 4C) and number (Fig. 4D) of donor-specific CTL in the spleen of host mice. The reduction in CTL was reflected in the higher percentage (Fig. 4E) and number (Fig. 4F) of host B cells remaining in the spleen compared with those in the vehicle- treated mice. The efficacy of daily treatment with 10-Cl-BBQ in suppressing GVHD was equivalent to the single dose of TCDD given on day 0.

### Structure-activity Studies with 10-Cl-BBQ Analogues

**Figure 5:**
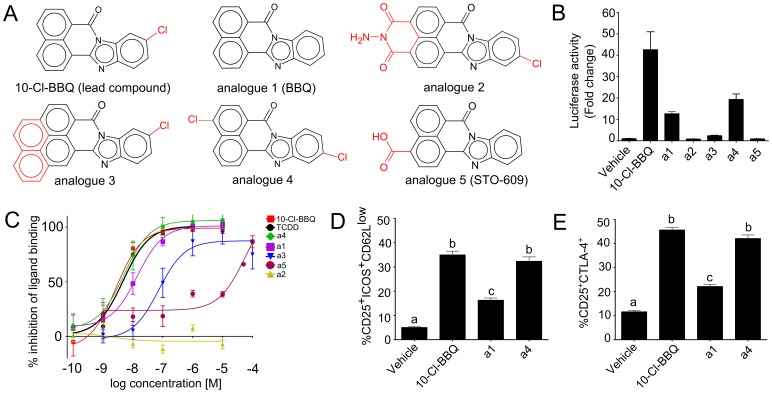
Structure activity studies with 10-Cl-BBQ analogues. **A)** Chemical structure of BBQ analogues with differences highlighted in red. See Table S2 for chemical names. **B)** Hepa1 cells expressing endogenous AhR were transfected with a XRE-luciferase reporter gene. The transfected cells were treated for 12 h with 10 nM of each compound (n = 3). The mean luciferase activity is shown with SEM. **C)** Dose response curves for the competitive inhibition of [^3^H] 3-MC binding to AhR. **D)** and **E)** Expression of phenotypic markers associated with AhR-Tregs in mice treated with BBQ analogues. Host mice were treated with 10 mg/kg of BBQ analogue or vehicle on day 0 and day 1 following donor cell transfer. On day 2, the alloactivated CD4^+^ donor T cells were identified based on CFSE dilution. The percentage of donor cells in each group expressing the Treg markers were derived from flow histograms. Data represent mean ± SEM of 5 mice per treatment; bars with different letter are significantly different, p<0.05.

Since 10-Cl-BBQ was identified in a screening program, it was of interest to determine if this specific BBQ derivative was uniquely potent as an AhR ligand. We tested five analogues of 10-Cl-BBQ (Fig. 5A, Table S2) including the unsubstituted BBQ, and a carboxylic acid-substituted BBQ, STO-609, which is used experimentally to inhibit Ca^2+^/Calmodulin-dependent protein kinase kinase (CaMKK) activity [26]. The analogues were tested at 10 nM for their ability to induce the AhR-regulated reporter gene activity. Analogues 1 (BBQ) and 4 (4,11-diCl-BBQ) induced 13- and 19-fold activation of AhR, respectively, while analogue 3 induced a modest 2-fold increase (Fig. 5B). Analogues 2 and 5 (STO-609) did not induce reporter activity at the 10 nM concentration tested.

The ligand binding affinity of each analogue was determined by their ability to competitively inhibit the binding of [^3^H] 3-methylcholanthrene (3-MC) to AhR (Fig. 5C). The concentration that inhibited binding by 50% (IC50) was calculated from the dose-response curves for each analogue and compared to the IC50 of 4.7 nM for TCDD. The IC50s of the lead compound (10-Cl-BBQ) and analogue 4 (4,11-diCl-BBQ) were similar to TCDD at 2.6 and 5.2 nM while the affinity of analogue 1 (BBQ) was lower at 13.7 nM. The IC50 of analogue 3 was 10-fold lower (77.3 nM), and analogue 5 (STO-609) was 1000-fold less active than TCDD at 45.2 µM. Analogue 2 was inactive over the concentration range tested.

Based on the higher binding affinity of analogue 1 and 4 compared to the other analogues, we selected these two compounds to determine if they were also capable of inducing Tregs during a GVH response. Compared with vehicle treatment, both compounds significantly increased the percentage of alloresponsive donor CD4^+^ T cells that expressed CD25 along with the AhR-Treg markers ICOS, CD62L^low^ (Fig. 5D) and CTLA-4 (Fig. 5E). Analogue 1 (BBQ) was significantly less potent compared to analogue 4 (4,11-diCl-BBQ) in inducing the Treg phenotype, while the potency of 4,11-diCl-BBQ was similar to 10-Cl-BBQ. These results are consistent with a structure-activity relationship (SAR) for Treg induction based on binding affinity of the ligand for AhR.

## Discussion

The overall objective of these studies was to identify high affinity AhR ligands with the ability to induce AhR-Tregs *in vivo* during a murine GVH response. Based on studies with TCDD, these AhR-Tregs are induced by direct activation of AhR in donor CD4^+^ T cells [4], [12] and differ from the Foxp3^+^ Tregs that are induced indirectly via AhR signaling in dendritic cells [3], [9]. Both types of Tregs likely contribute to the suppression of autoimmune and allergic diseases that has been reported following treatment with TCDD [4], [5], [15], [16], [27] as well as other AhR ligands [3], [28]. The ability of AhR ligands to directly induce antigen-specific Tregs via AhR activation in antigen-responsive CD4^+^ T cells, would bypass many of the barriers associated with current approaches used to enhance Treg function that rely on infusion of *ex vivo* expanded cells [18].

Here we describe the successful identification and characterization 10-Cl-BBQ, a potent AhR agonist that induces AhR-Tregs and suppresses murine GVHD. It has a short half-life with no acute toxicity at the dose required for its therapeutic effect, thereby making it a potential candidate for drug development. We also identified unsubstituted BBQ and 4,11-dichloro-BBQ as AhR ligands that are capable of inducing Tregs *in vivo*. In comparison to the unsubstituted BBQ, the addition of one or two chlorine atoms significantly increased the potency of the BBQ molecule to bind AhR and induce Tregs. The overlapping gene profiles induced by TCDD and 10-Cl BBQ in lymphocytes suggest that both compounds induce similar conformational changes in the AhR that are required for transcriptional activity. The planar conformation of both TCDD and 10-Cl-BBQ may account for these similarities [23].

STO-609 is a carboxylic acid substituted BBQ that is used experimentally to inhibit Ca^2+^/calmodulin-dependent protein kinase kinase (CaMKK) activity [26]. We found that STO-609 was a weak AhR agonist with an IC50 of 45 µM and thus we did not pursue its ability to induce Tregs. However, in previous studies designed to determine the role of Ca^2+^ in AhR signaling, STO-609 was discovered to be a weak, albeit full AhR agonist in MCF-7 cells and in human macrophages [29]. Interestingly, AhR activation occurred at the same concentration (25 µM) that was known to fully inhibit CaMKK [26], opening up the possibility that some of the effects attributed to CaMKK inhibition could be due to changes in expression of AhR-regulated genes. Like TCDD, STO-609 triggered increased [Ca^2+^] and activated CaMKIα [30]. Furthermore, since differential activation of the Ca^2+^/calmodulin-regulated transcription factor NFAT has been shown to be critical for T cell effector and regulatory functions [31], the role of this pathway in AhR-Treg induction deserves further study.

The characterization of 10-Cl-BBQ as a potent Treg-inducing AhR ligand is a promising step in the development of novel AhR-targeted therapeutics for treatment of immune-mediated diseases. Unfortunately, many pharmaceutical companies have excluded development of AhR ligands from their pipelines [32] due to concerns that CYP1A1 induction will increase cancer risk or otherwise induce “TCDD-like toxicity”. However, inadvertently, there are a large number of drugs on the market today that activate AhR and induce CYP1A1 as well as other AhR target genes in common with TCDD [20], [22], [33], [34]. These drugs have good safety records and are not known to increase cancer risk or produce chloracne, the most definitive sign of TCDD toxicity in humans. Some of these drugs show high affinity binding to AhR, but unlike TCDD, are metabolized and excreted [23]. A good example of the safe use of AhR ligands in humans is coal tar, a treatment for atopic dermatitis and psoriasis that has been used for more than 2000 years [35], [36]. Coal tar is composed of a highly complex mixture of organic chemicals that has been shown to induce AhR-regulated P450 enzymes, including Cyp1a1, in the skin following topical treatment [37], and coal tar metabolites are found in urine indicating systemic absorption [38]. However, in several studies, no increase in skin cancer or other malignancies has been noted in psoriasis patients treated with coal tar, including two 25-year follow-up trials [39]. Interestingly, the quinoline and isoquinoline fractions of coal tar were singled out thirty years ago as potential anti-psoriatic agents [40]. Furthermore, BBQs are known to be present in coal tar [41] and, as such, may be important contributors to the resolution of atopic dermatitis and psoriasis. Our studies suggest that the effectiveness of coal tar may be due, at least in part, to the induction of AhR-Tregs by the BBQ fraction.

In summary, the induction of Tregs *in vivo* by treatment with AhR ligands represents a new therapeutic approach for treatment of immune-mediated diseases. Tregs can be induced by targeting the AhR directly in CD4^+^ T cells [4], or indirectly via AhR activation in tolerogenic dendritic cells [42], [43]. Our data show that 10-Cl-BBQ directly targets CD4^+^ T cells to induce AhR-dependent Tregs while simultaneously suppressing murine GVHD without overt toxicity. Together with historical and structure-activity data, our study provides strong support for further clinical development of the BBQ class of compounds for use in AhR-Treg-based therapy.

## Supporting Information

Figure S1 **10-Cl-BBQ induces the expression of known AhR target genes.** Hepa1 cells were treated with 10-Cl-BBQ (10 nM), TCDD (10 nM), or vehicle (DMSO) for eight hours. RNA was extracted and RT-qPCR was performed for a select set of known AhR target genes. AHRR: aryl hydrocarbon receptor repressor, ALDH3A1: aldehyde dehydrogenase family 3; subfamily A1, CYP1b1: cytochrome P450 1b1; Gstm3: glutathione S-transferase, mu 3; GST-Ya: glutathione S transferase, alpha 1; NQO1: NAD(P)H dehydrogenase quinone 1; UGT2b34: UDP glucuronosyltransferase 2 family, polypeptide B34.
(TIF)

Figure S2 **10-Cl-BBQ does not inhibit T cell proliferation** ***in vitro*** **or** ***in vivo*** **.** **A.** Splenocytes from C57Bl/6 mice were labeled with CFSE and activated *in vitro* with anti-CD3 and anti-CD28 in the presence of 100 nM 10-Cl-BBQ or DMSO for 72 h. **B.** C57Bl/6 donor T cells were labeled with CFSE and injected into B6D2F1 host mice to initiate the GVH response. Host mice (n = 5 per group) were treated with vehicle or 10-Cl-BBQ i.p. (10 mg/kg/d) for two days. Donor cells were identified by gating on CD4^+^CFSE^+^ cells. Dilution of CFSE fluorescence demonstrates division of CD4^+^ T cells.
(TIF)

Figure S3 **Gating strategy for identifying alloactivated donor CD4^+^ T cells.** B6D2F1 host mice were injected with CFSE-labeled C57Bl/6 T cells on day 0 and given 10-Cl-BBQ, TCDD or vehicle by i.p. injections. At 48 h after GVH initiation, splenocytes were harvested from the host mice and the CD4^+^ T cells were identified by flow cytometry. Donor CFSE^+^ cells were gated on CD4^+^ T cells (rectangular region drawn in left histogram) and the alloactivated population was identified by CFSE dilution (rectangular region drawn in right histogram). The CFSE negative population represent host CD4^+^ cells.
(TIF)

Table S1 **Serum liver chemistry** **profile.** B6D2F1 mice were give 10 mg/kg/day of 10-Cl-BBQ or vehicle i.p. After 2 days serum was collected and analyzed. n = 5 mice per treatment, average values are shown(± standard deviation). ALT – Alanine transaminase, SGPT – serum glutamic pyruvate transaminase.
(DOCX)

Table S2 **Chemical names of analogues of 10-Cl-BBQ that were tested for AhR binding activity.**
(DOCX)
