# Supplementary figures and images for: Benzimidazoisoquinolines: A New Class of Rapidly Metabolized Aryl Hydrocarbon Receptor (AhR) Ligands that Induce AhR-Dependent Tregs and Prevent Murine Graft-Versus-Host Disease
Source: PLoS One. 2014 Feb 19;9(2):e88726. doi: 10.1371/journal.pone.0088726 (PMC3929365; doi:10.1371/journal.pone.0088726)

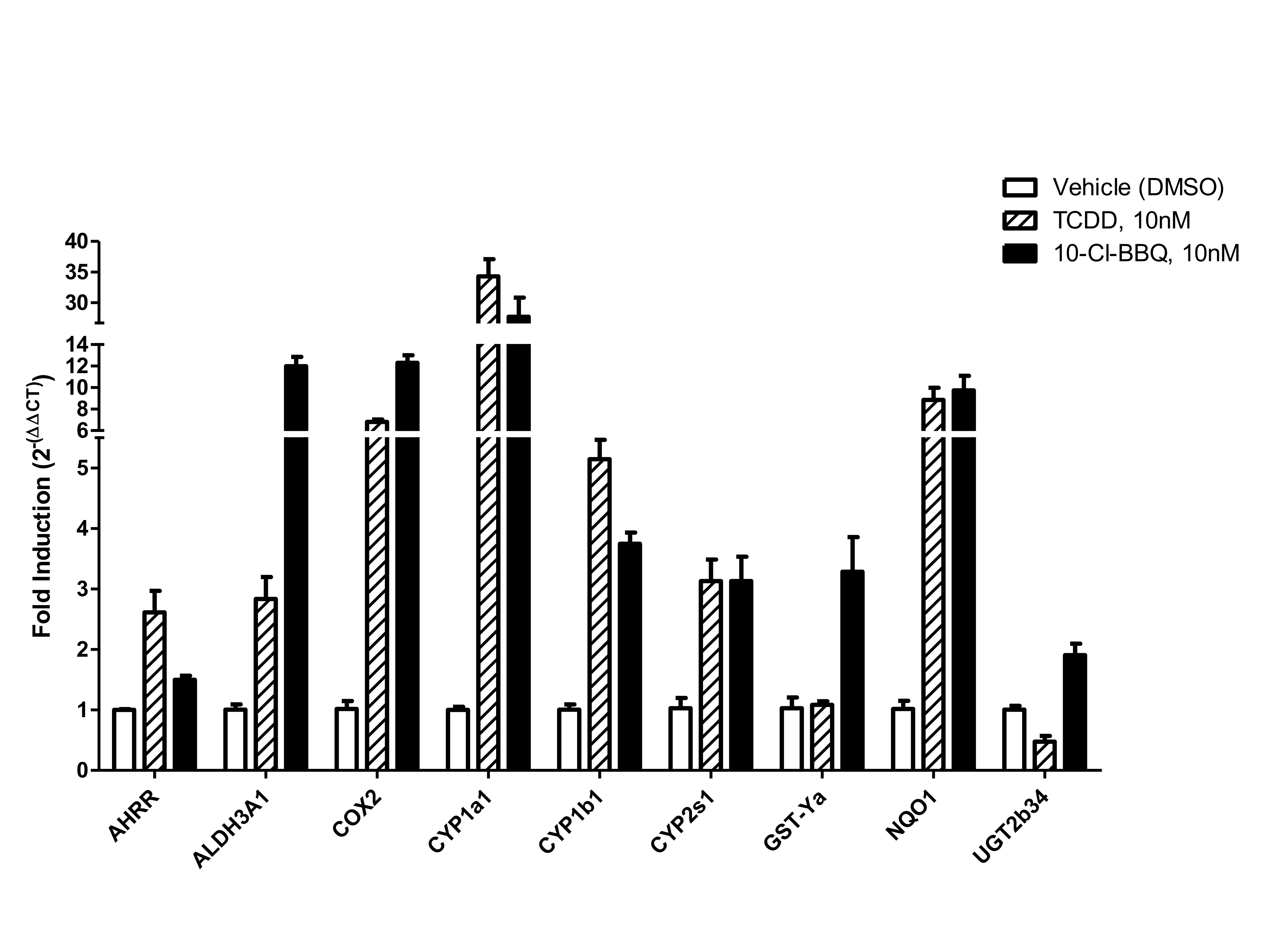

Supplement: Figure S1 — 10-Cl-BBQ induces the expression of known AhR target genes. Hepa1 cells were treated with 10-Cl-BBQ (10 nM), TCDD (10 nM), or vehicle (DMSO) for eight hours. RNA was extracted and RT-qPCR was performed for a select set of known AhR target genes. AHRR: aryl hydrocarbon receptor repressor, ALDH3A1: aldehyde dehydrogenase family 3; subfamily A1, CYP1b1: cytochrome P450 1b1; Gstm3: glutathione S-transferase, mu 3; GST-Ya: glutathione S transferase, alpha 1; NQO1: NAD(P)H dehydrogenase quinone 1; UGT2b34: UDP glucuronosyltransferase 2 family, polypeptide B34. (TIF) [file pone.0088726.s001.tif]

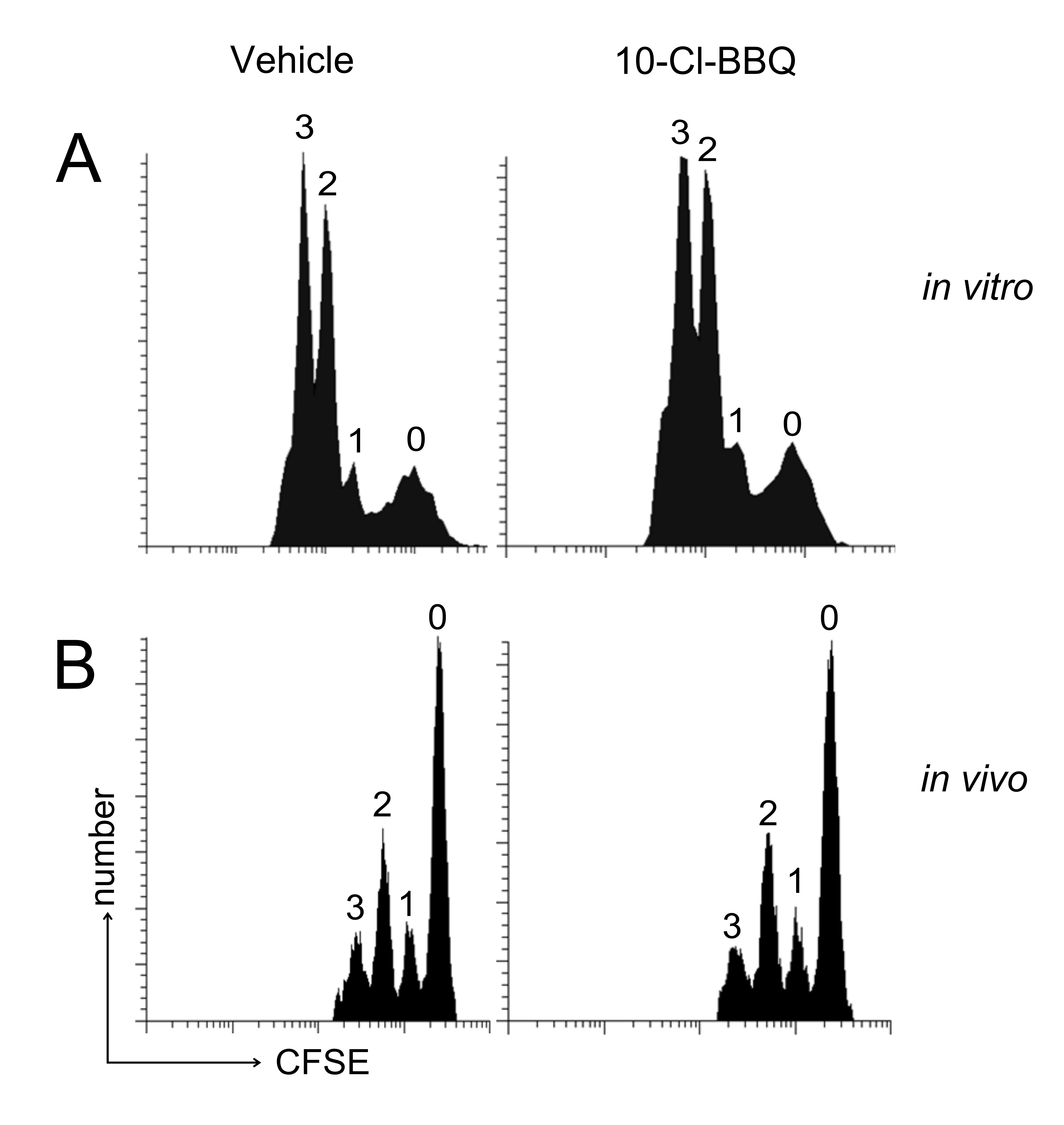

Supplement: Figure S2 — 10-Cl-BBQ does not inhibit T cell proliferation in vitro or in vivo . A. Splenocytes from C57Bl/6 mice were labeled with CFSE and activated in vitro with anti-CD3 and anti-CD28 in the presence of 100 nM 10-Cl-BBQ or DMSO for 72 h. B. C57Bl/6 donor T cells were labeled with CFSE and injected into B6D2F1 host mice to initiate the GVH response. Host mice (n = 5 per group) were treated with vehicle or 10-Cl-BBQ i.p. (10 mg/kg/d) for two days. Donor cells were identified by gating on CD4+CFSE+ cells. Dilution of CFSE fluorescence demonstrates division of CD4+ T cells. (TIF) [file pone.0088726.s002.tif]

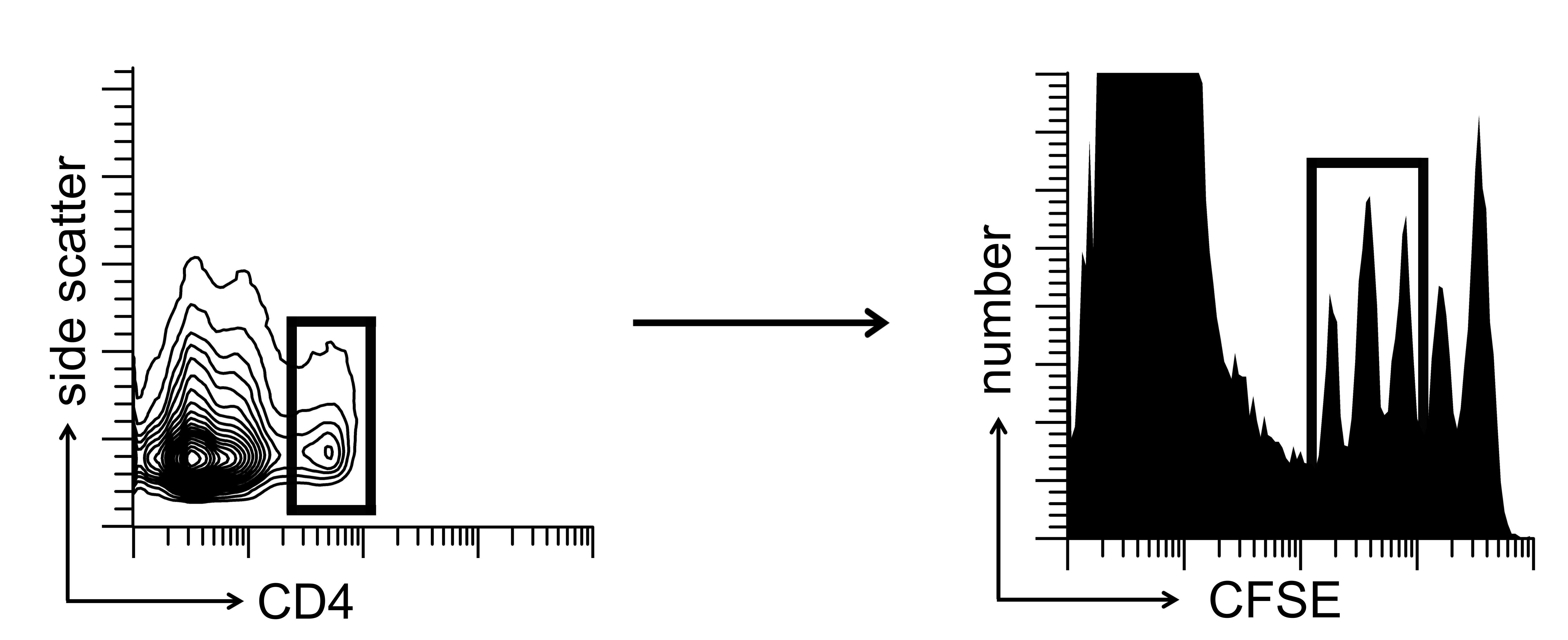

Supplement: Figure S3 — Gating strategy for identifying alloactivated donor CD4+ T cells. B6D2F1 host mice were injected with CFSE-labeled C57Bl/6 T cells on day 0 and given 10-Cl-BBQ, TCDD or vehicle by i.p. injections. At 48 h after GVH initiation, splenocytes were harvested from the host mice and the CD4+ T cells were identified by flow cytometry. Donor CFSE+ cells were gated on CD4+ T cells (rectangular region drawn in left histogram) and the alloactivated population was identified by CFSE dilution (rectangular region drawn in right histogram). The CFSE negative population represent host CD4+ cells. (TIF) [file pone.0088726.s003.tif]
