# Supplementary material for: Benzimidazoisoquinolines: A New Class of Rapidly Metabolized Aryl Hydrocarbon Receptor (AhR) Ligands that Induce AhR-Dependent Tregs and Prevent Murine Graft-Versus-Host Disease
Source: PLoS One. 2014 Feb 19;9(2):e88726. doi: 10.1371/journal.pone.0088726 (PMC3929365; doi:10.1371/journal.pone.0088726)
Supplement: Table S1 — Serum liver chemistry profile. B6D2F1 mice were give 10 mg/kg/day of 10-Cl-BBQ or vehicle i.p. After 2 days serum was collected and analyzed. n = 5 mice per treatment, average values are shown(± standard deviation). ALT – Alanine transaminase, SGPT – serum glutamic pyruvate transaminase. (DOCX) [file pone.0088726.s004.docx]

**Table S1. Serum liver chemistry** **profile**. B6D2F1 mice were give 10 mg/kg/day of 10-Cl-BBQ or vehicle i.p. After 2 days serum was collected and analyzed. n = 5 mice per treatment, average values are shown(± standard deviation). ALT – Alanine transaminase, SGPT – serum glutamic pyruvate transaminase.

| **Serum liver chemistry** | **Units** | **10-Cl-BBQ** | **Vehicle** | **Normal range** |
| --- | --- | --- | --- | --- |
| Blood Urea Nitrogen | mg/dl | 10.2 (± 1.5) | 12.2 (± 1.9) | \| 10-50 \| \| --- \| |
| Glucose | mg/dl | 201.2 (± 10.4) | 190.4 (± 30.4) | 30 - 250 |
| Cholesterol | mg/dl | 119.2 (± 10.4) | 105.8 (± 6.8) | 40 - 160 |
| Albumin | g/dl | 3.3 (± 0.1) | 3.3 (± 0.1) | 2.9 – 5.4 |
| Bilirubin | mg/dl | 0.12 (± 0) | 0.12 (± 0) | 0.1 – 1.2 |
| Alkaline Phosphatase | U/l | 51.4 (± 8.3) | 56.2 (± 2.6) | 36 - 300 |
| Gamma Glutamyl Transferase | U/l | 0 | 0 | - |
| ALT (SGPT) | U/l | 34.4 (± 10.4) | 35.2 (± 22.2) | 40 - 140 |
