# Supplementary material for: Benzimidazoisoquinolines: A New Class of Rapidly Metabolized Aryl Hydrocarbon Receptor (AhR) Ligands that Induce AhR-Dependent Tregs and Prevent Murine Graft-Versus-Host Disease
Source: PLoS One. 2014 Feb 19;9(2):e88726. doi: 10.1371/journal.pone.0088726 (PMC3929365; doi:10.1371/journal.pone.0088726)
Supplement: Table S2 — Chemical names of analogues of 10-Cl-BBQ that were tested for AhR binding activity. (DOCX) [file pone.0088726.s005.docx]

**Table S2. Chemical names of analogues of 10-Cl-BBQ that were tested for AhR binding activity.**

| **Analogue** | **IUPAC** | **other name** |
| --- | --- | --- |
| analogue 1  (BBQ) | 3,10-diazapentacyclo[10.7.1.0²,¹⁰.0⁴,⁹.0¹⁶,²⁰]icosa-1(20),2,4,6,8,12,14,16,18-nonaen-11-one | 7H-Benzimidazo[2,1-a]benzo[de]isoquinolin-7-one |
| analogue 2 | 17-amino-6-chloro-3,10,17-triazahexacyclo[13.6.2.0²,¹⁰.0⁴,⁹.0¹²,²².0¹⁹,²³]tricosa-1(21),2,4,6,8,12(22),13,15(23),19-nonaene-11,16,18-trione | 2-Amino-10-chlorobenzimidazo[2,1-b]benzo[lmn][3,8]phenanthroline-1,3,6(2H)-trione |
| analogue 3 | 7-chloro-3,10-diazaheptacyclo[20.3.1.0²,¹⁰.0⁴,⁹.0¹²,²⁵.0¹⁵,²⁴.0¹⁸,²³]hexacosa-1(26),2,4,6,8,12(25),13,15,17,19,21,23-dodecaen-11-one | 11-Chloro-8H-benzimidazo[2,1-a]phenanthro[3,4,5-defg]isoquinolin-8-one |
| analogue 4 | 6,15-dichloro-3,10-diazapentacyclo[10.7.1.0²,¹⁰.0⁴,⁹.0¹⁶,²⁰]icosa-1(19),2,4,6,8,12(20),13,15,17-nonaen-11-one | 4,11-Dichloro-7H-benzimidazo[2,1-a]benzo[de]isoquinolin-7-one |
| analogue 5 (STO-609) | 11-oxo-3,10-diazapentacyclo[10.7.1.0²,¹⁰.0⁴,⁹.0¹⁶,²⁰]icosa-1(20),2,4,6,8,12,14,16,18-nonaene-17-carboxylic acid | 7-Oxo-7H-benzimidazo[2,1-a]benzo[de]isoquinoline-3-carboxylic acid |
